# COVID-19, lifestyle behaviors and mental health: A mixed methods study of women 6 months following a hypertensive pregnancy

**DOI:** 10.3389/fpubh.2022.1000371

**Published:** 2022-10-18

**Authors:** Megan L. Gow, Chris Rossiter, Lynne Roberts, Maddison J. Henderson, Lin Yang, Judith Roche, Erin Hayes, Alison Canty, Elizabeth Denney-Wilson, Amanda Henry

**Affiliations:** ^1^The University of Sydney Children's Hospital Westmead Clinical School, Westmead, NSW, Australia; ^2^Discipline of Paediatrics and Child Health, School of Clinical Medicine, University of New South Wales, Sydney, NSW, Australia; ^3^Women's and Children's Health, St George Hospital, Kogarah, NSW, Australia; ^4^Susan Wakil School of Nursing and Midwifery, The University of Sydney, Sydney, NSW, Australia; ^5^St George and Sutherland Clinical Campus, School of Clinical Medicine, University of New South Wales, Sydney, NSW, Australia; ^6^Office of Medical Education, University of New South Wales, Sydney, NSW, Australia; ^7^Royal Hospital for Women, Randwick, NSW, Australia; ^8^Royal Prince Alfred Hospital, Camperdown, NSW, Australia; ^9^Women's Health Initiative Translational Unit, South Western Sydney Local Health District, Liverpool, NSW, Australia; ^10^Sydney Local Health District, Sydney, NSW, Australia; ^11^Discipline of Women's Health, School of Clinical Medicine, University of New South Wales, Sydney, NSW, Australia

**Keywords:** COVID-19, postpartum, diet, physical activity, depression, anxiety, hypertensive pregnancy, preeclampsia

## Abstract

**Introduction:**

The COVID-19 pandemic introduced unprecedented challenges to both the physical and psychological health of postpartum women. The aim of this study was to determine how the COVID-19 pandemic affected the diet, physical activity and mental health of women 6 months following a hypertensive disorder of pregnancy.

**Methods:**

Mixed methods sub-study of the Blood Pressure Postpartum trial, which recruited women following a hypertensive disorder of pregnancy from six Sydney metropolitan hospitals. Cross sectional analysis of baseline quantitative data, collected at 6-months postpartum from March 2019-February 2022, and qualitative data analysis from semi-structured telephone interviews, was performed. Dates of COVID-19 lockdowns for Sydney, Australia were collected from government websites. Diet (vegetable, fruit, alcohol, take away intake) and physical activity (walking, vigorous activity, strength training frequency and duration) were assessed using the self-report NSW Population Health Survey. Depression and anxiety were assessed using the Edinburgh Depression Scale and GAD-7 scale, respectively. Outcome data were compared between women who completed surveys “In Lockdown” vs. “Not in Lockdown” as well as “Prior to any Lockdown” vs. “During or Following any Lockdown”.

**Results:**

Of 506 participants, 84 women completed the study surveys “In Lockdown,” and 149 completed the surveys “Prior to any Lockdown.” Thirty-four participants were interviewed. There were no statistically significant differences in diet, physical activity, depression and anxiety among women who completed the survey “In Lockdown” vs. “Not in Lockdown.” “Prior to any Lockdown,” participants were more likely to do any walking (95% vs. 89%, *p* = 0.017), any vigorous activity (43% vs. 30%, *p* = 0.006) or any strength training (44% vs. 33%, *p* = 0.024), spent more time doing vigorous activity (*p* = 0.003) and strength training (*p* = 0.047) and were more likely to drink alcohol at least monthly (54% vs. 38%, *p* < 0.001) compared with “During or Following any Lockdown.”

**Conclusions:**

Our findings suggest that the confinements of lockdown did not markedly influence the mental health, diet and physical activity behaviors of women 6 months following hypertensive pregnancy. However, physical activity levels were reduced following the emergence of COVID-19, suggesting targeted efforts may be necessary to re-engage postpartum women with exercise.

**Trial registration:**

https://anzctr.org.au/Trial/Registration/TrialReview.aspx?id=376286&isReview=true, identifier: ACTRN12618002004246.

## Introduction

Hypertensive disorders of pregnancy (HDP) are associated with a myriad of short- and long-term health risks (1). Long-term vascular health is affected including a doubling of the lifetime risk of cardiovascular disease (CVD) and a three to four times increased risk of chronic hypertension within 15 years of HDP (2, 3). The 2022 International Society for the Study of Hypertension in Pregnancy guidelines recognize the importance of a healthy lifestyle in mitigating these risks, advising that, following a HDP, women receive education regarding the importance of maintaining healthy diet and physical activity behaviors long-term (1).

A more immediate health risk is the increased risk of clinically significant depressive and anxiety symptoms following a HDP (4). Preeclampsia specifically is an independent risk factor for both the incidence and the severity of anxiety and depressive symptoms (5). These perinatal mental illnesses have potentially long-term psychiatric and physical consequences for mother, child and the family as a whole (6).

In line with research demonstrating that disasters have a negative impact on perinatal mental and physical health (7), the COVID-19 pandemic may exacerbate mental health symptoms, and make adhering to healthy lifestyle behaviors more difficult. Several studies have reported that anxiety and depressive symptoms have increased in the perinatal population following the onset of the COVID-19 pandemic (8–10). Research reporting the effects of the pandemic on postpartum diet and physical activity are more limited. One study in Eastern Mediterranean postpartum women demonstrated poor adherence to US dietary guidelines overall, with modest increases in adherence since the onset of the pandemic (11). Similar findings were published by the same group in pregnant women (12). Other reports of the effect of the pandemic on diet are mixed, with studies in Ireland and Spain suggesting that eating patterns in pregnant women remained unchanged (13, 14), while one large cross-national study, also in pregnant women, reported significant adverse impacts on multiple health domains including diet, fitness and sleep (15). One other study also in a pregnant population suggested a significant decrease in physical activity levels at the onset of pandemic-related isolation (16).

There is limited research investigating the effects of COVID-19 on the lifestyle behaviors of postpartum women and no studies have investigated the impact of COVID-19 on the mental health and lifestyle behaviors of women following a HDP. It is critical to understand the impact of COVID-19 on the health of this vulnerable population due to the known short- and long-term risks of HDP which could be exacerbated by the pandemic (17, 18).

Therefore, the primary aim of this study was to determine the impact of COVID-19 on the diet, physical activity and mental health (depression and anxiety) of women at 6 months postpartum, following a HDP. Secondarily, this study aimed to determine how findings differed by location in metropolitan Sydney.

## Methods

This mixed-methods cross sectional study analyses data collected as part of the wider Blood Pressure Postpartum (BP^2^) research. BP^2^ is a three-arm randomized, multicentre trial currently underway at six metropolitan hospitals in Sydney, Australia (Campbelltown Hospital, Liverpool Hospital, Royal Hospital for Women, Royal Price Alfred Hospital, St George Hospital and Westmead Hospital). These hospitals include a broad sociodemographic spread of patient characteristics and draw from areas that experienced more vs. less strict COVID-19 lockdowns. The BP^2^ study protocol has been published (19). In brief, eligible participants were women aged ≥18 years who gave birth within the previous 6 months at one of the study hospitals and experienced a HDP. Women were excluded if they had known unavailability for follow-up, an active severe mental health condition, or developmental disability precluding informed consent.

Eligible women consenting to participate in BP^2^ complete their baseline questionnaires at 6 months (± 1 month) postpartum. They are then randomized to one of three groups:

1) optimized usual care: information package and family doctor follow-up 6 months postpartum;
2) brief intervention: information package as per group 1, plus assessment and brief lifestyle behavior change (LBC) counseling at a specialized clinic with an obstetric physician and dietitian 6 months postpartum; or
3) extended intervention: as per group 2 plus enrolment into a 6-month telephone-based LBC coaching program from 6 to 12 months postpartum.

The primary outcome measures of the BP^2^ study are (1) blood pressure (BP) change and (2) weight change and/or waist circumference change from 6 to 12 months postpartum. The BP^2^ study is powered to detect a 4 mmHg difference in systolic BP between groups, or a 4 kg weight loss difference/2cm waist circumference change.

### COVID-19 lockdowns in Sydney, Australia

For this study, “COVID-19 lockdown” was defined as any period in which stay-at-home orders were implemented by the New South Wales (NSW) Government because of the COVID-19 pandemic. Key lockdown dates in Sydney, Australia between March 2019 (BP^2^ study commencement) and February 2022 (end of data collection for this sub-study) are summarized in Supplementary Table S1.

Several Local Government Areas (LGAs) in Western and South-Western Sydney were subject to additional restrictions throughout the June to October 2021 lockdown due to high COVID-19 transmission rates in these areas (20). These came into force for eight LGAs on 28/07/2021 (Blacktown, Campbelltown, Canterbury-Bankstown, Cumberland, Fairfield, Georges River, Liverpool, and Parramatta LGAs) and in another four LGAs during August 2021 {Bayside, Burwood, Penrith [specific suburbs only (21)], and Strathfield LGAs} at which time these LGAs became known as the “12 LGAs of concern.” These more severe restrictions are outlined in Supplementary Table S2.

### Data collection

Quantitative data for this sub-study were collected at the BP^2^ study baseline, (6 months ± 1 month postpartum) and includes data from all consenting participants recruited from the date of BP^2^ study commencement in March 2019 until 22 February 2022 with baseline diet, physical activity, depression and/or anxiety data available. Qualitative data were collected by interviews conducted at 10–12 months postpartum with consenting women between March 2020 and April 2021; interviews were audio recorded and professionally transcribed.

Quantitative data were entered by research midwives onto Research Electronic Data Capture (REDCap) software. Demographic information included the participant's age, ethnicity and postcode. The postcode data were used to determine which LGA participants resided in at the time of completion of baseline BP^2^ questionnaires (22) and therefore whether they were in an “LGA of concern.” The ^**^timestamp^**^ feature on REDCap or date of study entry was used to determine whether each participant completed questionnaires “In Lockdown” or “Not in Lockdown” and “Prior to any Lockdown” or “During or Following any Lockdown”, as per Supplementary Table S1.

#### Lifestyle behavior

Diet and physical activity were assessed by self-report using selected questions from the NSW Population Health survey (23) which was administered by study midwives by phone at 6 months postpartum. Data collected included average vegetable serves per day; average fruit serves per day; average number of take away meals per month; average number of alcoholic drinks consumed each week; average time spent walking per week; average time spent doing vigorous physical activity per week; and average time spent doing strength training per week.

#### Depression

Depressive symptoms were assessed using the paper-based Edinburgh Postnatal Depression Scale (EPDS) (24), a validated and reliable (internal consistency coefficient, alpha = 0.92) self-rating scale questionnaire developed to screen depression postpartum (25, 26). The questionnaire consists of 10 statements, each scored on a four-point scale, rating the intensity of depressive symptoms present in the past week. A higher sum of score, reflects elevated severity of depressive symptoms with a range of zero to 30 (24). There is no universal consensus on the score used to diagnose postpartum depression. For the present analysis, a score ≥ 11 was used to indicate the presence of concern for major depression, as suggested by a 2020 systematic review to maximize sensitivity and specificity (27).

#### Anxiety

The presence and severity of generalized anxiety disorder (GAD) was assessed using the validated and reliable (internal consistency coefficient, alpha = 0.89) GAD-7 screening tool (28–30). It comprises seven items that describe prominent features of generalized anxiety, such as, excessive worry and irritability. Responses are scored from 0–3 with a possible total score of 0–21. Cut off scores of 5, 10, and 15 represent mild, moderate and severe anxiety levels, respectively (30, 31). In this study, a score of 10 or greater was used as the threshold for significant symptoms.

#### Qualitative data

Details regarding the qualitative data collection have been previously reported (32). In brief, all BP^2^ participants were invited to complete an optional telephone interview when their infants were approximately 10 months old. Interested participants received information about the qualitative sub-study and consent forms. Consenting participants were then contacted and a convenient time was arranged for their interview. Semi-structured interviews, ranging in length from 18 to 47 min, were conducted *via* telephone and audio-recorded with participants' consent by one interviewer [CR]. Interviews took place approximately 10 months postpartum. The schedule was informed by the Social Ecological Model (33) which recognizes that behavior is determined by the multi-layered and inter-connected effects of personal and environmental factors. The interview thus addressed barriers and facilitators to healthy eating and physical activity related to each layer: individual (knowledge, attitudes and behaviors), relationship, community, and societal factors.

All interviews were conducted from March 2020 to April 2021 which included periods of government stay-at-home orders across Sydney (Supplementary Table S1). As such, most women highlighted COVID-19 as being relevant to their health behavior and their experience on the BP^2^ study.

### Data analysis

Statistical analyses were performed using IBM SPSS Statistics, v27. Participants included in our analysis were (a) characterized using descriptive statistics, and (b) compared two ways (“In Lockdown” vs. “Not in Lockdown” and “Prior to any Lockdown” vs. “During or Following any Lockdown”) using independent sample *t*-tests or Mann Whitney U Tests (continuous variables) and Chi-square tests (categorical variables) as appropriate. Similarly, we conducted analysis to determine whether outcomes differed in participants located in an LGA of concern compared with those not located in an LGA of concern.

Qualitative data were uploaded to NVivo software v12 (QSR International Pty Ltd) for management and coding. For this sub-study, comments related to COVID-19 were identified and extracted from the interview transcripts by the interviewer. Interviews were not categorized by whether or not they were conducted during lockdowns, as participants described their experiences across the postnatal period, rather than at a single timepoint (or “during the last week”). De-identified extracts are included to provide depth to the findings from the quantitative analysis.

## Results

**Figure 1:**
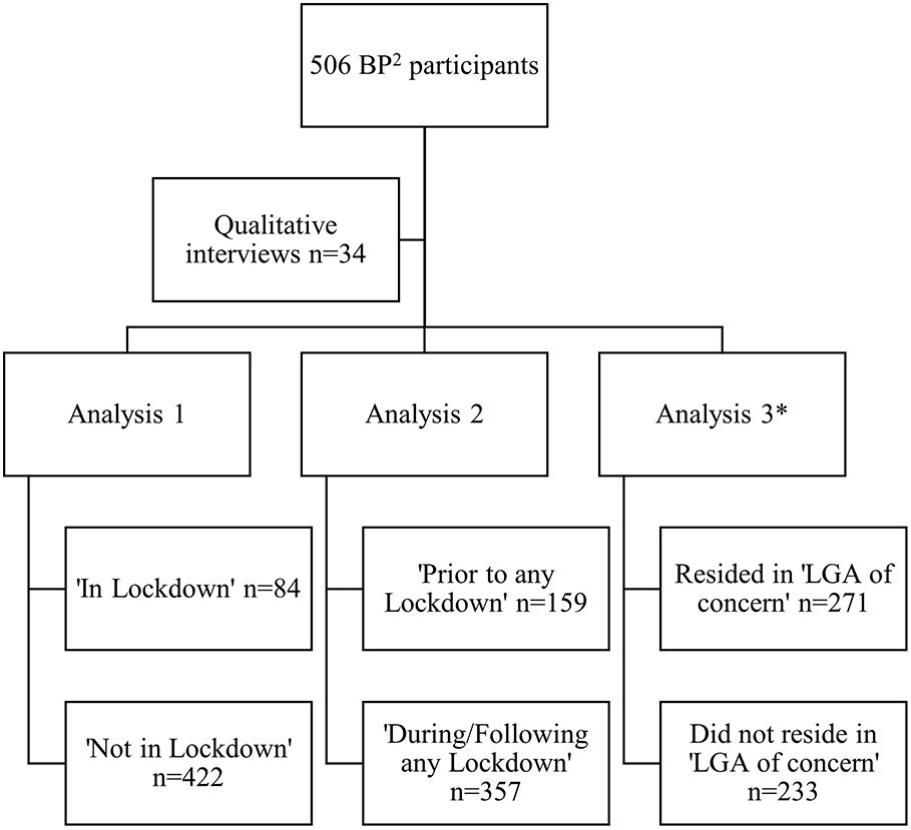
Flow chart of analysis conducted as part of this sub-study comparing the mental health and lifestyle behaviors of BP^2^ study participants at 6 months postpartum. ^*^Data on LGA of residence missing for two participants. BP^2^, Blood Pressure Postpartum study; LGA, local government area; n, number.

Quantitative data was available for 506 participants as part of this sub-study. Of these, 81 gave birth at St George Hospital, 175 at The Royal Hospital for Women, 74 at Royal Prince Alfred Hospital, 20 at Westmead Hospital, 85 at Campbelltown Hospital and 71 at Liverpool Hospital. Of the 506 participants, 84 women completed the study surveys during a government imposed COVD-19 lockdown, and 149 women completed the surveys prior to any government imposed COVD-19 lockdown. The remainder completed the surveys between lockdowns or after the last lockdown in October 2021. Thirty-four women participated in the semi-structured interviews. Their demographic characteristics were largely typical of the wider study population, albeit with higher proportions of both Australian-born and first-time mothers (32). The various analyses conducted as part of this sub-study are outlined in Figure 1.

For the first analysis, the characteristics of women who completed the 6-month study surveys “In Lockdown” compared with those who were “Not in Lockdown” are outlined in Table 1. All demographic data were similar between groups, as were diet, physical activity and mental health outcomes.

**Table 1:** Characteristics of women at 6-months postpartum participating in the BP^2^ study who filled in study questionnaires during a COVID-19 lockdown vs. those not in a lockdown.

	**In lockdown** **(*n* = 84)**	**Not in lockdown** **(*n* = 422)**	***P*** **–value**
Age (at time of giving birth), years	33.51 ± 5.29	33.81 ± 5.19	0.637
Booking–in BMI, kg/m^2^	27.23 ± 6.65	26.97 ± 6.36	0.738
Weight status, *n (%)*			0.709
Underweight	1 (1)	11 (3)	
Normal weight	37 (45)	181 (44)	
Overweight	21 (26)	122 (30)	
Obesity	23 (28)	100 (24)	
Ethnicity, *n (%)*			0.595
Caucasian	51 (71)	288 (74)	
Asian	12 (17)	73 (19)	
Aboriginal/ Torres Strait Islander	1 (1)	3 (1)	
Other	8 (11)	27 (7)	
Hypertensive disorder of pregnancy:			0.097
Chronic hypertension, *n (%)*	17 (20)	59 (14)	
Gestational hypertension, *n (%)*	14 (17)	113 (27)	
Preeclampsia, *n (%)*	45 (54)	226 (54)	
Preeclampsia/ chronic hypertension, *n (%)*	8 (10)	24 (6)	
Gestational diabetes, *n (%)*	13 (16)	68 (16)	0.884
EPDS score, median [IQR]	5.5 [2.5–8.5]	5.0 [2.0–8.0]	0.463
Depression (EPDS score ≥ 11), *n (%)*	13 (17)	62 (15)	0.735
GAD−7 score, median [IQR]	3.0 [0.5–5.5]	2.0 [0.0–4.0]	0.138
Anxiety (GAD−7 ≥ 10), *n (%)*	5 (6)	34 (8)	0.574
Time spent walking, minutes per week, median [IQR]	150 [30–270]	180 [75–285]	0.352
Any walking, *n (%)*	75 (89)	381 (91)	0.684
Time spent in vigorous physical activity, minutes per week, median [IQR]	0.0 [−15.0–15.0]	0.0 [−26.8–26.8]	0.342
Any vigorous physical activity, *n (%)*	25 (30)	146 (35)	0.423
Time spent doing strength training, minutes per week, median [IQR]	0.0 [−15.0–15.0]	0.0 [−22.5–22.5]	0.998
Any strength training, *n (%)*	30 (36)	153 (36)	0.925
Vegetable serves per day, median [IQR]	2.0 [1.0–3.0]	2.0 [1.0–3.0]	0.429
Meeting recommended 5/d vegetables, *n (%)*	13 (9)	31 (9)	0.978
Fruit serves per day, median [IQR]	1.0 [−0.7–2.3]	1.0 [0.5–1.5]	0.261
Meeting recommended 2 serves fruit per day, *n (%)*	34 (41)	190 (45)	0.474
Take away occasions, per month, median [IQR]	3.0 [1.35–4.65]	2.0 [0.35–3.65]	0.866
Alcohol, drinks per week, median [IQR]	0.0 [−1.0–1.0]	0.0 [−0.85–0.85]	0.921
Drink alcohol at least monthly, *n (%)*	36 (43)	179 (43)	0.954

Missing data: BMI and weight status, n = 10; ethnicity, n = 43; EPDS, n = 19; GAD, n = 18; walking and vigorous physical activity, n = 2; vegetables serves, n = 2; fruit serves, n = 3; take away occasions, n = 5; alcohol intake, n = 3.

BMI, body mass index; BP^2^, Blood Pressure Postpartum; d, day; EPDS, Edinburgh Postnatal Depression Scale; GAD, generalized anxiety disorder; IQR, interquartile range; kg, kilogram; m, meter; min, minutes; n, number; wk, week.

The second analysis compared women who completed the 6-month study surveys “Prior to any Lockdown” compared with those who completed the surveys at any time “During or Following any Lockdown” (from 31 March 2020), outlined in Table 2. Demographics were similar between groups. However, participants were more likely to do any walking, any vigorous activity or any strength training, spent more time doing vigorous activity and strength training and were more likely to drink alcohol at least monthly prior to any lockdown compared with those surveyed during or following any lockdown. Of note, 11% percent and 5% of women met the threshold for depression and anxiety respectively “Prior to any Lockdown,” which increased to 17 and 9% respectively “During or Following any Lockdown.” Further, 12% screened positive for either depression or anxiety “Prior to any Lockdown” compared with 19% “During or Following any Lockdown” (*p* = 0.056).

**Table 2:** Characteristics of women at 6–months postpartum participating in the BP^2^ study who filled in study questionnaires prior to any COVID−19 lockdown vs. those during or following any COVID−19 lockdown.

	**Prior to any lockdown (*n* = 149)**	**During/ following any lockdown (*n* = 357)**	***P*** **–value**
Age (at time of giving birth), years	34.01 ± 5.10	33.65 ± 5.24	0.474
Booking–in BMI, kg/m^2^	26.53 ± 5.90	27.22 ± 6.60	0.272
Weight status, *n (%)*			0.544
Underweight	3 (2)	9 (3)	
Normal weight	70 (48)	148 (42)	
Overweight	36 (25)	107 (31)	
Obesity	37 (25)	86 (25)	
Ethnicity, *n (%)*			0.205
Caucasian	112 (79)	227 (71)	
Asian	22 (16)	63 (20)	
Aboriginal/ Torres Strait Islander	0 (0)	4 (1)	
Other	8 (6)	27 (8)	
Hypertensive disorder of pregnancy:			0.088
Chronic hypertension, *n (%)*	17 (11)	59 (17)	
Gestational hypertension, *n (%)*	48 (32)	79 (22)	
Preeclampsia, *n (%)*	75 (50)	196 (55)	
Preeclampsia/ chronic hypertension, *n (%)*	9 (6)	23 (6)	
Gestational diabetes, *n (%)*	25 (17)	56 (16)	0.760
EPDS score, median [IQR]	4.0 [1.0–7.0]	5.0 [2.0–8.0]	0.057
Depression (EPDS score ≥ 11), *n (%)*	16 (11)	59 (17)	0.069
GAD−7 score, median [IQR]	2.0 [0.5–3.5]	2.0 [0.0–4.0]	0.304
Anxiety (GAD−7 ≥ 10), *n (%)*	7 (5)	32 (9)	0.084
Time spent walking, minutes per week, median [IQR]	210 [120–300]	179 [51–307]	0.111
**Any walking**, ***n (%)***	**142 (95)**	**314 (89)**	**0.017**
**Time spent in vigorous physical activity, minutes per week, median [IQR]**	**0.0 [−42.5–42.5]**	**0.0 [−15.0–15.0]**	**0.003**
**Any vigorous physical activity**, ***n (%)***	**64 (43)**	**107 (30)**	**0.006**
**Time spent doing strength training, minutes per week, median [IQR]**	**0.0 [−30.0–30.0]**	**0.0 [−20.0–20.0]**	**0.047**
**Any strength training**, ***n (%)***	**65 (44)**	**118 (33)**	**0.024**
Vegetable serves per day, median [IQR]	2.0 [0.8–3.3]	2.0 [1.0–3.0]	0.617
Meeting recommended 5/d vegetables, *n (%)*	5 (6)	39 (9)	0.323
Fruit serves per day, median [IQR]	1.0 [0.5–1.5]	1.0 [0.5–1.5]	0.625
Meeting recommended 2 serves fruit per day, *n (%)*	68 (46)	156 (44)	0.681
Take away occasions, per month, median [IQR]	2.0 [0.0–4.1]	3.0 [1.35–4.65]	0.204
**Alcohol, drinks per week, median [IQR]**	**0.23 [−0.77–1.23]**	**0.0 [−0.5–0.5]**	**0.002**
**Drink alcohol at least monthly**, ***n (%)***	**81 (54)**	**134 (38)**	**< 0.001**

Missing data: BMI and weight status, n = 10; ethnicity, n = 43; EPDS, n = 19; GAD, n = 18; walking and vigorous physical activity, n = 2; vegetables serves, n = 2; fruit serves, n = 3; take away occasions, n = 5; alcohol intake, n = 3.

BMI, body mass index; BP^2^, Blood Pressure Postpartum; d, day; EPDS, Edinburgh Postnatal Depression Scale; GAD, generalized anxiety disorder; IQR, interquartile range; kg, kilogram; m, meter; min, minutes; n, number; wk, week. Bold values indicate significant differences between groups.

Data on LGA of residence were available for 504 participants: 271 lived in an “LGA of concern” and 233 did not. Overall, participants who resided in an LGA of concern were younger and more likely to have overweight/ obesity, less likely to be Caucasian, more likely to have a pregnancy also complicated by gestational diabetes, and to have done little/no physical activity. They also did less walking, vigorous physical activity and strength training, consumed fewer vegetables and fruit, consumed more take away meals, and consumed less alcohol (Table 3). There were no differences in mental health outcomes.

**Table 3:** Characteristics of women at 6–months postpartum participating in the BP^2^ study who lived in a Local Government Area of concern vs. those who did not live in a Local Government Area of concern at time of study entry^*^.

	**LGA of concern** **(*n* = 271)**	**Not LGA of concern** **(*n* = 233)**	***P*** **–value**
Age (at time of giving birth), years	**33.3 ±5.5**	**34.3 ±4.7**	**0.026**
Booking–in BMI, kg/m^2^	**27 8 ±6.8**	**26.1 ±5.8**	**0.002**
Weight status, *n (%)*			**0.001**
Underweight	**9 (3)**	**3 (1)**	
Normal weight	**96 (36)**	**121 (53)**	
Overweight	**83 (31)**	**60 (26)**	
Obesity	**77 (29)**	**45 (20)**	
Ethnicity, *n (%)*			**<0.001**
Caucasian	**142 (60)**	**196 (87)**	
Asian	**60 (26)**	**24 (11)**	
Aboriginal/ Torres Strait Islander	**2 (1)**	**2 (1)**	
Other	**31 (13)**	**4 (2)**	
Hypertensive disorder of pregnancy:			0.080
Chronic hypertension, *n (%)*	18	12	
Gestational hypertension, *n (%)*	22	28	
Preeclampsia, *n (%)*	53	55	
Preeclampsia/ chronic hypertension, *n (%)*	7	5	
**Gestational diabetes**, ***n (%)***	**52 (19)**	**29 (12)**	**0.040**
EPDS score, median [IQR]	5.0 [2.0–8.0]	5.0 [1.5–8.5]	0.677
Depression (EPDS score ≥ 11), *n (%)*	41 (16)	33 (14)	0.624
GAD−7 score, median [IQR]	2.0 [0.0–4.0]	2.0 [0.0–4.0]	0.376
Anxiety (GAD−7 ≥ 10), *n (%)*	25 (10)	14 (6)	0.143
**Time spent walking, minutes per week, median [IQR]**	**120 [5–335]**	**210 [60–360]**	**<0.001**
**Any walking**, ***n (%)***	**228 (85)**	**226 (97)**	**<0.001**
**Time spent in vigorous physical activity, minutes per week, median [IQR]**	**0 [−240–240]**	**0.0 [−34–34]**	**<0.001**
**Any vigorous physical activity**, ***n (%)***	**66 (25)**	**105 (45)**	**<0.001**
**Time spent doing strength training, minutes per week, median [IQR]**	**0 [−8–8]**	**0 [−30–30]**	**<0.001**
**Any strength training**, ***n (%)***	**73 (27)**	**104 (45)**	**<0.001**
**Vegetable serves per day, median [IQR]**	**2.0 [1.0–3.0]**	**3.0 [2.0–4.0]**	**<0.001**
Meeting recommended 5/d vegetables, *n (%)*	24 (9)	20 (9)	0.916
**Fruit serves per day, median [IQR]**	**1.0 [0.3–1.7]**	**1.5 [1.0–2.0]**	**0.012**
Meeting recommended 2 serves fruit per day, *n (%)*	114 (42)	110 (48)	0.226
**Take away occasions, per month, median [IQR]**	**3.0 [1.3–4.7]**	**2.0 [−0.2–4.2]**	**0.045**
**Alcohol, drinks per week, median [IQR]**	**0.0 [−0.2–0.2]**	**1.0 [−0.5–2.5]**	**< 0.001**
**Drink alcohol at least monthly**, ***n (%)***	**74 (27)**	**140 (60)**	**<0.001**

* Two participants did not give postcode data so are excluded from this analysis.

Missing data: BMI and weight status, n = 10; ethnicity, n = 43; EPDS, n = 19; GAD, n = 18; walking and vigorous physical activity, n = 2; vegetables serves, n = 2; fruit serves, n = 3; take away occasions, n = 5; alcohol intake, n = 3.

BMI, body mass index; BP^2^, Blood Pressure Postpartum; d, day; EPDS, Edinburgh Postnatal Depression Scale; GAD, generalized anxiety disorder; IQR, interquartile range; LGA, Local Government Area; kg, kilogram; m, meter; min, minutes; n, number; wk, week. Bold values indicate significant differences between groups.

Our final analysis looked at only those participants who lived in an LGA of concern and compared outcomes in the 53 participants who completed surveys “Prior to any Lockdown” (pre-March 2020) with the 45 participants who completed surveys during or following the 2021 lockdown (from 28/07/2021) when restrictions were heightened for those who lived in an LGA of concern. In this analysis there were no significant differences between groups in physical activity or dietary behaviors, or in mental health outcomes (Supplementary Table S3).

### Qualitative results

Although not a specific question, most women mentioned the COVID-19 pandemic during the interviews. Participants identified both positive and negative impacts on their health behaviors and broader lifestyle. None of the interviewees reported having contracted COVID-19 (the last interviews were in April 2021 prior to widespread community transmission in Sydney), but most highlighted the disruption to their lives from stay-at-home orders and border closures. Some reported difficulties in accessing health services for themselves or their babies (including attending study sites for 6-month assessments and, where relevant, consultations with health professionals as part of the intervention).

No one's been going to the GP for just routine check-ups recently. So I haven't been for a couple of months. (#08, Group 1)

Conversely, those in intervention group 3 found telephone-based contact with a healthy lifestyle coach convenient.

Certainly in the last little while [during lockdown] it's been more convenient than going somewhere to see someone. It's a lot easier. (#05, Group 3)

Several women recounted the negative impact on their physical activity, including the closure of gyms, swimming pools and other facilities just as they were planning to return to regular exercise. However, nearly all women mentioned that they walked regularly with their babies. This fulfilled a dual need for physical activity and social contact. During lockdowns, walking and outside exercise were among the few ways to legitimately spend time with others from outside their household. Some mentioned that online platforms enabled them to take up training or yoga without the need for childcare arrangements or the cost of gym membership, possibly returning to regular physical activity sooner than they might have done without lockdowns.

I probably got to the age now where I would have been going back and doing more swims and stuff, but obviously all the swimming pools are closed. (#08, Group 1)

So after they closed the gyms, the exercising has been a lot, lot less. We still try to go for walks, but we're doing the best in the situation we can. (#09, Group 2)

My gym does have a creche. I just haven't felt comfortable about putting her there yet with COVID and everything. So, I've kind of strayed away from that. (#29, Group 2)

I would just walk the dogs around the streets. That's all we could do really. (#18, Group 2)

But the good thing I think about the coronavirus situation is there's a lot of content online and a lot of studios are putting stuff up online for free. (#04, Group 3)

I'm limited I guess somewhat in leaving him. I mean COVID's kind of been a good thing for me because the, um, the online stuff is actually really suiting my personal situation. (#05, Group 3)

With the coronavirus, it's meant that people can actually join us for a walk and get to see the twins. Because before it was more my anxiety about bringing infection into the home, because I've got, a lot of our family are either in teaching positions or in healthcare. (#12, Group 2)

Even with COVID happening, I had no idea there were so many young families or young mums in my area than when all of a sudden they all came out of the woodwork during COVID and you saw them walking around. (#14, Group 1)

Most participants' comments regarding diet indicated a negative impact. Several women reported being unable to buy fresh food due to lockdowns, increased costs and a greater consumption of unhealthy take-away meals and junk food. Working from home sometimes led to increased snacking and some women reported increasedalcohol consumption.

I probably wasn't getting as much fresh fruit and veg as I was before because I just wasn't venturing out because of her. I wasn't risking any of it. (#14, Group 1)

And now that I'm working from home, the kitchen's nearby. (#15, Group 1)

So I was doing my click and collect online shopping and all that stuff, like I just put a lot of chocolates and stuff in there (#19, Group 3)

I definitely think being in this Corona lockdown I'm drinking more… [Previously] I wouldn't drink during the week and I just have wine usually with dinner on the weekends. But I feel like every night I'm like a glass of wine, a couple of glasses of wine. (#04, Group 3)

Conversely a few participants mentioned embracing healthy cooking as a family lockdown activity.

We make our own chicken stock. Grow our own herbs. Yeah, it's kind of like our hobby. (#03, Group 2)

We love to cook. We cook most of our food from scratch, we don't do a lot of takeaways or sauces, packet mix or that type of thing (#04, Group 3)

The pandemic also had a profound effect on interpersonal relations. Some participants with partners welcomed them working from home to have more “family time,” and to share childcare, enabling the women to exercise. Others, however, reported friction or financial stress related to unemployment. Some mothers reported loneliness or isolation, including single parents or recent immigrants. Many felt cut off from support networks, especially if friends and family were interstate or overseas and unable to travel.

So my husband's working from home a lot. I think that's definitely helping out in terms of me being able to attend a particular class. (#32, Group 3)

One of the good things about corona virus—obviously he's been working from home for last few months. And I think he's finally realized actually what it's like, how long the days are… So I think he's a bit more aware now that there's no time for myself, which I don't think he'd properly computed before. (#08, Group 1)

It was good in the fact that my husband and I got to spend so much time with my daughter in those very early stages. (#17, Group 3)

Coronavirus has ruined all of these things. My sister-in-law lives across the road from us. So normally I guess I could have been, can you just come in and look after him for an hour? But she's gone back to her parents' house for the duration of coronavirus in Queensland. (#08, Group 1)

Another concern was the closure of community-based “mothers' groups,” usually facilitated by early childhood health centers and a frequent source of companionship, advice and mutual support for new parents. Although some had social media contact with other mothers, others felt this was a lost opportunity.

I sort of was looking forward to having a little mother's group with [baby] and we didn't really get to do it because it just kind of–the pandemic hit. (#24, Group 1)

Several women noted the impact on their psychosocial well-being, highlighting the isolation, stress and uncertainty of experiencing the pandemic in early parenthood.

I would say that the lockdown completely changed everything as well… Maybe I would be in more of a routine if we were... we didn't have to go through that... I didn't want to try and be healthy at that point. (#11, Group 2)

Well, it started off good. And then just walking and that, and then it went downhill again because of COVID. That's why I stacked on the weight. I wasn't getting out, I wasn't doing anything. (#19, Group 3)

I went to the mother's group two times… but then it was all closed up… I always try every day to go out somewhere with the baby, but there isn't anymore like the library, I don't know…. So, I definitely do a lot of googling that I know that you shouldn't do. But what to do? (#22, Group 2).

## Discussion

To the best of our knowledge, this is the first study to combine quantitative and qualitative data regarding the impact of COVID-19 on the mental health, diet and physical activity behaviors of women at 6-months postpartum. Our quantitative findings suggest that the confinements of lockdown did not influence the mental health, diet and physical activity behaviors of women following a HDP. However, there were challenges following the commencement of COVID-19 in Australia whereby women were more active prior to any COVID-19 related lockdown compared with anytime from 31 March 2020 when COVID-19 stay-at-home laws were first introduced in Sydney. The qualitative data highlight some of the impacts of the pandemic on mental health and physical wellbeing.

Our study did not find a significant difference in mental health outcomes reported by women by COVID-19 lockdown status, in contrast to findings from survey studies conducted in countries including the US, Canada, Mexico, Belgium, Italy and Qatar, all of which report heightened postpartum depressive and anxiety symptoms (8, 9, 34–37) during the local peak of COVID-19 compared to pre-COVID. To our knowledge there has only been one qualitative study (38), conducted in Australia, in which semi-structured interviews with postpartum women supported the findings of increased depressive and anxiety symptoms reported in previous quantitative studies. Although not statistically significant, women in our study meeting the threshold for significant depression symptoms increased and anxiety symptoms almost doubled during or following any lockdown. This is potentially clinically relevant, and lack of statistical significance may be due to lack of power, not lack of difference.

We found reduced exercise levels following the onset of any COVID-19 lockdown. These findings were supported by our qualitative data which indicated that gym closures, reduced access and/or unwillingness to utilize childcare options, and working from home arrangements all impacted the ability of women to partake in formal or organized physical activity. Similar experiences were reported in the qualitative study by Lim et al., also in postpartum women (38). This reduction in activity levels could signify potential ramifications of the pandemic on the physical health of women following a HDP. Targeted approaches may be necessary to reengage women with exercise post-pandemic to prevent long-term consequences of poor activity levels, especially for women following HDP who are susceptible to poor vascular health outcomes.

Despite our overall findings of reduced activity following any COVID-19 related lockdown, our qualitative data showed that many women utilized COVID-19 restrictions to support new exercise behaviors, including walking as a way to socialize with family and friends with reduced risk of viral transmission—or simply to “get out of the house.” These qualitative data highlight the individual variation in response to the pandemic. Similarly, our qualitative data demonstrated individual variation regarding impacts on diet whereby some reported unavailability of fresh food and reduced shopping opportunities as a barrier to improved diet whereas others took the view that, more time spent at home meant more time to prepare meals, and fewer opportunities for eating out and buying takeaway meant that diet improvements were easier to maintain. These variations in responses highlighted by our qualitative data may be reflected in our quantitative data where we saw no differences in dietary intake at a group level. Future analyses of BP^2^ data will examine other predictors of diet and physical activity behaviors of women in this study including demographic, cardiometabolic and mental health outcomes.

Our findings regarding consumption of alcohol are interesting. Our qualitative data contrasted with our quantitative findings, whereby our quantitative findings suggested reduced intake “During or Following any Lockdown” compared with “Prior to any Lockdown” whereas, in interviews, some women reported drinking more alcohol in light of the pandemic. It is possible though, that the reduced ability to socialize (social drinks) in combination with the reduced intake of alcohol among many of the women during pregnancy and into early parenthood may have removed the expected effect of the pandemic on alcohol intake among our cohort. Furthermore, as qualitative interviews were conducted 10–12 months postpartum (as opposed to quantitative data which were collected at 6 months postpartum) it is also possible that reduced or discontinued breastfeeding may have contributed to increased alcohol consumption, for enjoyment or as a coping mechanism, by this time.

In areas where more stringent restrictions were enforced during the 2021 stay-at-home orders, women displayed a vastly different demographic and lifestyle profile compared with those from areas where stay-at-home orders were not as severe. They typically engaged less frequently, and overall spent less time, doing physical activity, and their diet represented a less healthy eating pattern. In those living in an LGA of concern we did not see any differences among participants when looking at their data before any lockdown vs. during or following these more stringent stay-at-home orders, suggesting that our findings may be related to the socioeconomic and demographic differences across Sydney, rather than simply a reflection of the differences in stay-at-home orders.

There are a number of strengths of the present study. Firstly, the inclusion of both quantitative and qualitative data provides a more nuanced view of how individuals and families react to stressors such as those experienced during the pandemic, enhancing the overall validity of our results. Our sample of 506 for the quantitative aspect of this study is also larger than most previous studies assessing the impact of COVID-19 on health outcomes in postpartum women. Despite these strengths there are also several limitations. Firstly, it relies on self-reported qualitative and quantitative data. As the surveys and interviews included questions relating to sensitive topics, such as mental health, dietary intake, alcohol consumption and physical activity levels, it is possible that the data have been affected by external bias resulting from social desirability or by recall bias. On 11/10/2021 lockdown ended for fully vaccinated individuals; however, we did not record who in our study sample was vaccinated/unvaccinated. This was not accounted for in analysis; however, with 70% of Sydney's adults fully vaccinated at this time (39), all participants were assumed to be out of lockdown from this date. Quantitative and qualitative data were collected at different time points (6 months vs. 10 months postpartum), so data are not truly comparable. However, all interviewees had experienced some form of lockdown, and participants brought up issues related to the pandemic freely, with some reflecting on periods of lockdown. In fact, our qualitative data showed the complexity and range of negative impacts of lockdowns which may not have emerged with a point-of-time comparison. Finally, data were unpaired and, as this study represents a secondary analysis of the broader BP^2^ study, we were not powered to find differences by time of data collection.

In conclusion, our findings suggest that the confinements of lockdown did not significantly influence the mental health, diet and physical activity behaviors of women 6 months following a HDP. However, physical activity levels were reduced following the emergence of COVID-19, suggesting targeted efforts may be necessary to reengage postpartum women with regular formal exercise sessions. Qualitative data highlight the individualized and far-reaching experience of COVID-19 suggesting the need to consider this when counseling as part of clinical practice.

## Data availability statement

The raw data supporting the conclusions of this article will be made available by the authors, without undue reservation.

## Ethics statement

The studies involving human participants were reviewed and approved by South-Eastern Sydney Local Health District Human Research Ethics Committee (Ref: 18/193, REGIS: 2019/ETH04732). The patients/participants provided their written informed consent to participate in this study.

## Author contributions

CR, LR, AC, JR, EH, and MH substantially contributed to acquisition of data. MG, CR, LR, ED-W, and AH contributed to the conception and design of the broader BP^2^ study and/or this sub-study. MG conducted the analysis. MG and CR contributed to interpretation of the data. MG, CR, MH, and LY drafted parts of the work. All authors read and approved the final manuscript and agree to be personally accountable for their contributions and will ensure that any questions related to the accuracy or integrity of any part of the work will be appropriately investigated, resolved, and the resolution documented in the literature.

## Funding

This study was funded by the NSW Health Translational Research Grants Scheme (Round 3 TRGS application 26). MG and AH's work on the study was supported by a National Health and Medical Research Council (Australia) Early Career Fellowship (APP 1158876 and GNT 1141570, respectively). The funding bodies had no role in the study design, data collection or analysis, nor in writing the manuscript.

## Conflict of interest

The authors declare that the research was conducted in the absence of any commercial or financial relationships that could be construed as a potential conflict of interest.

## Publisher's note

All claims expressed in this article are solely those of the authors and do not necessarily represent those of their affiliated organizations, or those of the publisher, the editors and the reviewers. Any product that may be evaluated in this article, or claim that may be made by its manufacturer, is not guaranteed or endorsed by the publisher.

## Abbreviations

BP^2^: Blood Pressure Postpartum
EPDS: Edinburgh Postnatal Depression Scale
GAD: generalized anxiety disorder
HDP: hypertensive disorder of pregnancy
LBC: lifestyle behavior change
LGA: Local Government Area
REDCap: Research Electronic Data Capture.
