# Supplementary material for: COVID-19, lifestyle behaviors and mental health: A mixed methods study of women 6 months following a hypertensive pregnancy
Source: Front Public Health. 2022 Oct 18;10:1000371. doi: 10.3389/fpubh.2022.1000371 (PMC9623114; doi:10.3389/fpubh.2022.1000371)
Supplement: Supplementary file 1 [file Table_1.DOCX]

Supplementary Material

**Supplementary Table 1.** Lockdown dates in New South Wales, Australia between 1^st^ January 2019 and 22^nd^ February 2022 for areas relevant to participants of the BP^2^ study

| **Date** | **Lockdown Areas** |
| --- | --- |
| 01/03/2019 – 30/03/2020 | Nil |
| 31/03/2020 – 14/05/2020 | All of NSW (1, 2) |
| 15/05/2020 – 18/12/2020 | Nil |
| 19/12/2020 – 08/01/2021 | Northern Beaches LGA (3, 4) |
| 09/01/2021 – 24/06/2021 | Nil |
| 25/06/2021 – 10/10/2021 | Sydney, Woollahra, Randwick, Waverley LGAs (5, 6)* |
| 26/06/2021 – 10/10/2021 | As above, plus Greater Sydney, Blue Mountains, Central Coast, Wollongong, and Shellharbour (6, 7)* |
| 11/10/2021 – 31/12/2021 | Nil** |
| 01/01/2022 – 22/02/2022 | Nil |

* Various levels of lockdown laws put in place depending on local government area of residence (see Additional File 1 for more details)

** Lockdown ended on 11/10/2021 only for fully vaccinated individuals

Abbreviations: BP^2^, Blood Pressure Postpartum; LGA, Local Government Area; NSW, New South Wales.

**References**

1. Public health orders relating to gathering and movement. NSW legislation. https://legislation.nsw.gov.au/information/covid19-legislation/gathering-and-movement. Accessed 11 March 2022.
2. NSW to ease COVID-19 restrictions from Friday 15 May [press release]. NSW Government. https://www.nsw.gov.au/news/nsw-to-ease-covid-19-restrictions-from-friday-15-may. Accessed 11 March 2022.
3. Public Health Order for Northern Beaches LGA. NSW Government. https://www.health.nsw.gov.au/news/Pages/20201219_03.aspx. Accessed 11 March 2022.
4. Update on restrictions 2 January 2021 [press release]. NSW Government. https://www.nsw.gov.au/media-releases/update-on-restrictions-2-january-2021. Accessed 11 March 2022.
5. Greater Sydney COVID-19 restrictions extended [press release]. NSW Government. https://www.nsw.gov.au/media-releases/greater-sydney-covid-19-restrictions-extended. Accessed 11 March 2022.
6. NSW freedoms never tasted so sweet [press release]. NSW Government. [https://www.nsw.gov.au/media-releases/nsw-freedoms-never-tasted-so-sweet. Accessed 11 March 2022](https://www.nsw.gov.au/media-releases/nsw-freedoms-never-tasted-so-sweet.%20Accessed%2011%20March%202022).
7. COVID-19 restrictions extended in NSW [press release]. NSW Government. https://www.nsw.gov.au/media-releases/covid-19-restrictions-extended-nsw. Accessed 11 March 2022.

**Supplementary Table 2.** Different lockdown laws for the ‘12 LGAs of concern’ in Sydney versus the rest of Greater Sydney from July to October 2021 relevant to BP^2^ study participants

| **Date law came into effect** | **Lockdown law for the ‘LGAs of concern’** | **Lockdown law for the rest of Greater Sydney** |
| --- | --- | --- |
| 28/07/2021 | By the end of July, residents in Blacktown, Campbelltown, Canterbury-Bankstown, Cumberland, Fairfield, Georges River, Liverpool, and Parramatta LGAs were only permitted to leave their LGA if they were an authorised worker (1) |  |
| 30/07/2021 | Residents in the above ‘8 LGAs of concern’ could only exercise and shop within 5km of their home (2) | Residents could only exercise and shop within their LGA or 10km of their home [introduced on 9^th^ July 2021 (exercise) (3) and 28^th^ July 2021 (shopping) (1) |
| 08/08/2021 – 12/08/2021 | By mid-August, Bayside, Burwood, Penrith (specific suburbs only (4)), and Strathfield LGAs were also identified as ‘areas of concern’ and were subject to the above restrictions (2) |  |
| 16/08/2021 | Residents were not permitted to gather outdoors for recreation (5) | Outdoor recreation was capped at two people, excluding those from the same household (introduced on 9^th^ July 2021) (3)  Additionally, from this date, residents could only exercise and shop within their LGA or 5km of their home (5) |
| 23/08/2021 | Residents of the ‘12 LGAs of concern’ were subject to additional restrictions, including a curfew from 9pm to 5am (excluding authorised workers, emergencies, or medical care), and one-hour limit on outdoor exercise per day (6) |  |
| 13/09/2021 | Households with all adults vaccinated could gather outdoors for recreation, within existing restrictions (i.e., between the hours of 5am and 9pm, for one hour only, and within 5km of their home). This was in addition to their one-hour of exercise (7) | Five people (including children and all adults had to be fully vaccinated) could gather outdoors within their LGA or 5km of their home (7) |
| 15/09/2021 | Curfews lifted in the ‘12 LGAs of concern’ (4) |  |
| 20/09/2021 | Most restrictions aligned with other lockdown areas, including: no time limit on outdoor exercise and recreation, up to five fully vaccinated adults could gather outdoors in their LGA or within 5km of their home, and residents could now exercise and shop within their LGA or 5km of their home (previously only the latter was permitted (8) |  |
| 11/10/2021 | Once 70% of eligible individuals in NSW were double vaccinated, several restrictions eased across the state for these people regardless of their area of residence, including larger gatherings in the home (10 fully vaccinated visitors) and outdoors (30 fully vaccinated people), and the re-opening of hospitality venues and gyms (9, 10) | |

Abbreviations: BP2, Blood Pressure Postpartum; LGA, Local Government Area

**References**

1. Certainty for the community as restrictions adjusted and vaccines ramped up. NSW Liberal. <https://nswliberal.org.au/Shared-Content/News/2021/Restrictions-adjusted-and-vaccines-ramped-up>. Accessed 30 March 2022.
2. Fighting the Delta outbreak with new restrictions for local government areas (LGAs) of concern. NSW Health. <https://www.health.nsw.gov.au/news/Pages/20210730_01.aspx>. Accessed 30 March 2022.
3. COVID-19 Update: Restrictions - 9 July 2021. NSW Liberal. <https://nswliberal.org.au/Shared-Content/News/2021/COVID-19-Update-Restrictions-9-July-2021>. Accessed 30 March 2022.
4. NSW lifts curfew in LGAs of concern. NSW Liberal. <https://nswliberal.org.au/Shared-Content/News/2021/NSW-lifts-curfew-in-LGAs-of-concern>. Accessed 30 March 2022.
5. Increased fines, test and isolate payments and new compliance measures as NSW battles Delta. NSW Liberal. <https://nswliberal.org.au/Shared-Content/News/2021/Increased-fines,-test-and-isolate-payments-and-new>. Accessed 30 March 2022.
6. New protections and compliance rules to carry NSW through to vaccination targets. NSW Liberal. <https://nswliberal.org.au/Shared-Content/News/2021/New-protections-and-compliance-rules-to-carry-NSW>. Accessed 30 March 2022.
7. New freedoms for vaccinated - first step on state roadmap out of COVID. NSW Liberal. <https://nswliberal.org.au/Shared-Content/News/2021/New-freedoms-for-vaccinated>. Accessed 30 March 2022.
8. Restrictions to ease in LGAs of concern. NSW Liberal. <https://nswliberal.org.au/Shared-Content/News/2021/Restrictions-to-ease-in-LGAs-of-concern>. Accessed 30 March 2022.
9. NSW freedoms never tasted so sweet [press release]. NSW Government. [https://www.nsw.gov.au/media-releases/nsw-freedoms-never-tasted-so-sweet. Accessed 11 March 2022](https://www.nsw.gov.au/media-releases/nsw-freedoms-never-tasted-so-sweet.%20Accessed%2011%20March%202022).
10. NSW on the road to reopening [press release]. NSW Liberal. <https://nswliberal.org.au/Shared-Content/News/2021/NSW-on-the-road-to-reopening>. Accessed 30 March 2022.

**Supplementary Table 3.** Sub-analysis of participants located in a Local Government Area of concern prior to any lockdown versus during or following the July to October 2021 lockdown

|  | Prior to any lockdown (n=53) | During/ following 2021 lockdown (n=45) | P value |
| --- | --- | --- | --- |
| Age (at time of giving birth), years | 33.9 ± 6.0 | 33.8 ± 5.4 | 0.924 |
| Booking-in BMI, kg/m^2^ | 27.3 ± 7.0 | 28.0 ± 6.4 | 0.112 |
| Weight status, n (%)  Underweight  Normal weight  Overweight  Obesity | 2 (4)  23 (45)  9 (18)  17 (33) | 1 (2)  14 (32)  18 (41)  11 (25) | 0.097 |
| Ethnicity, n (%)  Caucasian  Asian  Aboriginal/ Torres Strait Islander  Other | 29 (60)  14 (29)  0 (0)  5 (10) | 26 (67)  6 (15)  0 (0)  7 (18) | 0.247 |
| Hypertensive disorder of pregnancy:  Chronic hypertension, n (%)  Gestational hypertension, n (%)  Preeclampsia, n (%)  Preeclampsia/ chronic hypertension, n (%) | 7 (13)  16 (30)  27 (51)  3 (6) | 9 (20)  5 (11)  27 (60)  4 (9) | 0.136 |
| Gestational diabetes, n (%) | 11 (21) | 8 (18) | 0.710 |
| EPDS score, median [IQR] | 4.0 [1.0-7.0] | 4.5 [1.0-8.0] | 0.363 |
| Depression (EPDS score ≥ 11), n (%) | 4 (8) | 7 (16) | 0.208 |
| GAD-7 score, median [IQR] | 2.0 [0.0-4.0] | 2.0 [-0.5-4.5] | 0.625 |
| Anxiety (GAD-7 ≥ 10), n (%) | 4 (8) | 4 (9) | 0.805 |
| Time spent walking, minutes per week, median [IQR] | 150 [65-235] | 165 [26-304] | 0.756 |
| Any walking, n (%) | 46 (89) | 36 (80) | 0.365 |
| Time spent in vigorous physical activity, minutes per week, median [IQR] | 0 [-8-8] | 0 [0] | 0.127 |
| Any vigorous physical activity, n (%) | 14 (26) | 6 (14) | 0.121 |
| Time spent doing strength training, minutes per week, median [IQR] | 0 [-15-15] | 0 [0] | 0.055 |
| Any strength training, n (%) | 16 (30) | 7 (16) | 0.089 |
| Vegetable serves per day, median [IQR] | 2.0 [2.0] | 2.0 [2.6] | 0.434 |
| Meeting recommended 5/d vegetables, n (%) | 4 (8) | 8 (18) | 0 124 |
| Fruit serves per day, median [IQR] | 1.0 [1.6] | 1.0 [1.2] | 0.541 |
| Meeting recommended 2 serves fruit per day, n (%) | 19 (36) | 18 (40) | 0.673 |
| Take away occasions, per month, median [IQR] | 2.0 [3.3] | 4.3 [3.3] | 0.891 |
| Alcohol, drinks per week, median [IQR] | 0.0 [0.5] | 0.0 [0.6] | 0.826 |
| Drink alcohol at least monthly, n (%) | 17 (32) | 13 (29) | 0.733 |
| Drink alcohol at least weekly, n (%) | 9 (17) | 7 (16) | 0.849 |

Missing data: BMI and weight status, n=3; ethnicity n=11; EPDS, n=2; GAD n=2; walking, vigorous physical activity and strength training n=2; vegetable serves, n=1; fruit serves n=1; take away occasions n=1; alcohol intake, n=2.

Abbreviations: BMI, body mass index; BP^2^, Blood Pressure Postpartum; d, day; EPDS, Edinburgh Postnatal Depression Scale; GAD, generalised anxiety disorder; IQR, interquartile range; kg, kilogram; m, metre; min, minutes; n, number; wk, week
